# Monocyte to High-Density Lipoprotein Ratio (MHR) and Monocyte to Lymphocyte Ratio (MLR) in Vitiligo: A Case-Control Study

**DOI:** 10.7759/cureus.93737

**Published:** 2025-10-02

**Authors:** Vaniyapilly S Aathira, Satyaki Ganguly

**Affiliations:** 1 Dermatology, All India Institute of Medical Sciences, Raipur, Raipur, IND

**Keywords:** lymphocyte, monocyte, neutrophil, oxidative stress, stability, vitiligo

## Abstract

Background

Vitiligo is a disorder influenced by oxidative stress and systemic inflammation. While previous studies used difficult-to-perform markers, research on readily available markers like the monocyte to high-density lipoprotein ratio (MHR), monocyte to lymphocyte ratio (MLR), platelet to lymphocyte ratio (PLR), and neutrophil to lymphocyte ratio (NLR) for oxidative stress in vitiligo remains limited, and assessing these markers in stable and unstable vitiligo represents a novel area of research.

Materials and methods

This case-control study was conducted on 72 vitiligo patients and an equal number of age and gender-matched healthy controls. Out of 72 vitiligo patients, 57 patients had unstable vitiligo and 15 patients had stable vitiligo. Complete blood count (CBC) and fasting lipid profile (FLP) were performed for both cases and controls to assess MHR, MLR, NLR, and PLR. Further, 19 patients on systemic therapy were reassessed after one month. Their ratios were compared before and after treatment.

Results

Vitiligo patients had significantly higher MHR, MLR, NLR, and PLR as compared to healthy controls. Compared to controls, stable vitiligo patients had no significant difference in these markers. Only PLR was significantly higher in unstable compared to stable vitiligo patients. There was no statistically significant reduction in these markers after one month of systemic treatment in 19 patients.

## Introduction

Vitiligo is a disorder of complex etiology. Among the theories proposed for the pathogenesis of vitiligo, the antioxidant-oxidant hypothesis, autoimmune theory, autocytotoxicity theory, and neural humoral hypothesis have gained wide attention.

Reactive oxygen species (ROS) reduce the formation of dendrites of melanocytes, weaken the adhesion of melanocytes to the basal layer, and cause their separation. ROS also increases the release of proinflammatory cytokines and accelerates the separation of melanocytes from the basal layer [1].

Multiple studies have investigated oxidative stress in vitiligo, utilizing indicators such as hydrogen peroxide, glutathione peroxidase, catalase, malondialdehyde, and superoxide dismutase [2-5]. Nevertheless, these indicators are not used routinely because of unavailability and cost.

Monocytes are considered an important source of pro-inflammatory factors that can lead to inflammation, thrombosis, and endothelial dysfunction. In contrast, high-density lipoprotein (HDL) cholesterol has anti-inflammatory, anti-oxidant, and anti-thrombotic effects [6]. Neutrophils release neutrophil elastase, matrix metalloproteinase, and cytokines. Lymphocytes have anti-inflammatory properties. Platelet release and activation are modulated by extracellular ROS in a microenvironment-dependent manner [7].

Therefore, the monocyte to HDL ratio (MHR), monocyte to lymphocyte ratio (MLR), platelet to lymphocyte ratio (PLR), and neutrophil to lymphocyte ratio (NLR) can be used as markers for inflammatory diseases [1]. These markers have been investigated in various systemic and dermatological diseases like psoriasis, Behcet’s disease, acne vulgaris, lichen planus, and hidradenitis suppurativa as markers of oxidative stress [8-12]. Since complete blood count (CBC) and fasting lipid profile (FLP) are simple, widely available tests, these markers could be simple means to assess the oxidative stress in vitiligo patients.

There is some evidence of the presence of oxidative stress in vitiligo patients. Confirmation of the oxidative stress as well as reduction, if any, in the markers for oxidative stress after treatment, can justify immunosuppressive and antioxidant therapy for vitiligo patients with active disease.

## Materials and methods

This was a case-control study including clinically diagnosed vitiligo patients more than 18 years of age presenting to the department of dermatology, All India Institute of Medical Sciences, Raipur, and an equal number of age and gender-matched healthy controls over a period of one and a half years, from 7/11/2022 to 10/5/2024, after obtaining approval from the institutional ethical committee. Patients and controls with known autoimmune or chronic inflammatory diseases, dyslipidemia, and those patients who have received systemic treatment or photototherapy in the last two months were excluded from this study. The diagnosis of vitiligo was made based on clinical examination, and features were assessed by dermoscopy.

The severity of the disease was assessed using the vitiligo extent tensity index (VETI) and stability by the vitiligo disease activity (VIDA) score [13,14].

The VIDA score was calculated as shown in Table 1.

**Table 1:** Calculation of the vitiligo disease activity (VIDA) score

Disease activity	VIDA Score
Active in the past 6 weeks/less duration	+4
Active in the past 6 weeks to 3 months	+3
Active in the past 3 months to 6 months	+2
Active in the past 6 months to 1 year	+1
Stable for at least 1 year or more	0
Stable for at least 1 year or more and spontaneous repigmentation	-1

Patients with VIDA scores of -1 and 0 were considered to have stable disease, and scores of +1, +2, +3, and +4 were considered to have unstable disease.

The VETI score was calculated as follows:

VETI score = (percentage of head involvement x stage) + (percentage of trunk involvement x stage) 4 + (percentage of upper extremity involvement x stage) 2 + (percentage of lower extremity x stage) 4 +(percentage of genital organ involvement x stage) 0.1.

The percentage of involvement is assessed using the rule of nines.

Stage of severity: Stage 0 - Normal skin; Stage 1 - Hypopigmentation; Stage 2 - Complete depigmentation with black hairs and perifollicular pigmentation; Stage 3 - Complete depigmentation with black hairs without perifollicular pigmentation; Stage 4 - Complete depigmentation with white and black hairs with or without perifollicular pigmentation; Stage 5 - Complete depigmentation with significant whitening in hairs.

A high VETI score indicates more severe and extensive vitiligo.

The stability of vitiligo was defined as no new lesions and no progression of existing lesions for at least one year. For both patients and controls, complete blood count (CBC) and fasting lipid profile (FLP) were performed to assess MHR, MLR, NLR, and PLR. Further, 19 vitiligo patients on systemic therapy were reassessed after one month, with repeat CBC and FLP. Their ratios were compared pre- and post-treatment.

Data analysis was done using the Statistical Package for Social Sciences (SPSS) software for Windows, v 22 (IBM Corp., Armonk, NY, US). Descriptive and two-tailed comparative tests, including the chi-squared and Fisher’s exact tests, were performed for categorical variables and Mann-Whitney tests for numerical variables, respectively, to compare vitiligo patients with controls. Correlation between the variables was done with Spearman’s rank correlation coefficient. A p-value of less than 0.05 was considered statistically significant.

## Results

A total of 72 clinically diagnosed vitiligo patients and an equal number of age and gender-matched healthy controls were included in the study. The majority, comprising 20 (27.8%) and 17 (23.6%) patients, fell within the age group of 21 to 30 years and 31 to 40 years, respectively. The mean age of the study subjects was 38.64 ± 14.67 years in cases and controls (the range was from 18 to 73 years) (Table 2).

**Table 2:** Comparison of age (years) between cases and controls ‡ Independent t-test, † Chi-squared test

Age (years)	Cases (n=72)	Controls (n=72)	Total	P value	Test statistical value
18 to 20 years	5 (6.94%)	5 (6.94%)	10 (6.94%)	1^†^	Chi-squared test -0
21 to 30 years	20 (27.78%)	20 (27.78%)	40 (27.78%)		
31 to 40 years	17 (23.61%)	17 (23.61%)	34 (23.61%)		
41 to 50 years	13 (18.06%)	13 (18.06%)	26 (18.06%)		
51 to 60 years	11 (15.28%)	11 (15.28%)	22 (15.28%)		
>60 years	6 (8.33%)	6 (8.33%)	12 (8.33%)		
Mean ± SD	38.64 ± 14.67	38.64 ± 14.67	38.64 ± 14.62	1^‡^	T-test - 0
Range	18-73	18-73	18-73		

In the case and control groups, 40 (55.56%) were males and 32 (44.44%) were females (male to female ratio of 1.25:1) (Table 3).

**Table 3:** Comparison of gender between cases and controls † Chi-squared test

Gender	Cases (n=72)	Controls (n=72)	Total	P value
Female	32 (44.44%)	32 (44.44%)	64 (44.44%)	1^†^
Male	40 (55.56%)	40 (55.56%)	80 (55.56%)	
Total	72 (100%)	72 (100%)	144 (100%)	

The duration of disease among the study subjects had a median value (25th-75th percentile) of 3 years (1-9). Analysis of systemic treatment history among the subjects revealed that alternative treatments were more commonly used, with Ayurvedic treatment being the most common, used by 31 patients (43.06%), followed by Homeopathic treatment by 11 patients (15.28%). In contrast, allopathic treatments were used less commonly, with systemic steroids used by 10 (13.89%), and both Methotrexate and Tofacitinib being used by one patient each (1.39%).

Among the vitiligo patients, 11 cases (15.28%) were known patients of hypothyroidism, 2 cases (2.78%) were diagnosed with hypothyroidism, and 1 patient (1.39%) was diagnosed with hyperthyroidism on further investigations. Family history of vitiligo was present in nine patients (12.50%), more commonly among the first-degree relatives of vitiligo patients.

The VIDA score was used to determine the stability of the disease. Patients with VIDA scores of -1 and 0 were considered to have stable disease, and the rest were considered to have unstable disease. According to this, 57 patients (79.17%) had unstable vitiligo, while 15 patients (20.83%) had stable vitiligo (Table 4).

**Table 4:** VIDA score distribution among vitiligo patients VIDA: vitiligo disease activity

VIDA score	Frequency (n=72)	Percentage
-1	5	6.94%
0	10	13.89%
1+	12	16.67%
2+	11	15.28%
3+	15	20.83%
4+	19	26.39%
Total	72	100.00%

Mean body surface area involvement was 5.17% ± 11.59. The majority of patients had more than one site involvement, and the most common site involved was the limbs (79.17%). Oral mucosal involvement was more common, present in 18 patients (25.00%), followed by both oral and genital mucosa in 7 (9.72%), and only genital mucosa in 4 patients (5.56%). The most common type of vitiligo seen was generalized in 47 patients (65.28%), followed by acral in 10 patients (13.89%) (Table 5).

**Table 5:** Type of vitiligo among patients

Type of vitiligo	Frequency	Percentage
Generalized	47	65.28%
Acral	10	13.89%
Acrofacial	6	8.33%
Focal	1	1.39%
Mucosal unclassified	5	6.94%
Segmental	2	2.78%
Universal	1	1.39%
Total	72	100.00%

On clinical examination, perifollicular repigmentation was observed in 53.33% of the stable patients compared to 12.28% of the unstable patients (p = 0.0005). Marginal repigmentation was present in 80% of the stable compared to 12.28% of the unstable patients (p < .0001) (Table 6).

**Table 6:** Association of clinical features with the stability of vitiligo * Fisher's exact test, † Chi-squared test

Clinical features	Stable(n=15)	Unstable(n=57)	P value	Test statistical value
Perifollicular repigmentation	8 (53.33%)	7 (12.28%)	0.0005^†^	Chi-squared value – 12.13
Marginal repigmentation	12 (80%)	7 (12.28%)	<.0001^*^	Fisher's exact test
Koebner phenomenon	0 (0%)	18 (31.58%)	0.015^*^	

Disease severity was assessed with the VETI score, and the mean value of VETI was 0.59 ± 1.24. A significant weak positive correlation was seen between the VIDA score and VETI (p value =.002).

Compared to controls, vitiligo patients had significantly higher MHR, MLR, NLR, and PLR (p value <.005) (Table 7).

**Table 7:** Comparison of MHR, MLR, NLR, and PLR between vitiligo patients and controls § Mann-Whitney test

MHR, MLR, NLR, PLR	Cases (n=72)	Controls (n=72)	P value	Test statistical value
Monocyte to high-density lipoprotein (HDL) ratio (MHR)				
Mean ± SD	0.01 ± 0.019	0.0043 ± 0.0036	<.0001^§^	1519.5
Range	0.001-0.06	0.001-0.02		
Monocyte to lymphocyte ratio (MLR)				
Mean ± SD	0.16 ± 0.14	0.07 ± 0.06	<.0001^§^	1358.5
Range	0.01-0.89	0.01-0.31		
Neutrophil to lymphocyte ratio (NLR)				
Mean ± SD	1.91 ± 1	1.37 ± 0.37	0.0002^§^	1654
Range	0.01-6.8	0.66-2.65		
Platelet to lymphocyte ratio (PLR)				
Mean ± SD	114.9 ± 44.86	87.83 ± 29.85	<.0001^§^	1535
Range	42.5-315	36.98-168.86		

While unstable vitiligo patients had a significantly higher MHR, MLR, NLR, and PLR compared to controls, stable vitiligo patients showed no significant difference in these values when compared to controls (Tables 8, 9).

**Table 8:** Comparison of MHR, MLR, NLR, and PLR between unstable vitiligo patients and age and gender-matched controls § Mann-Whitney test

MHR, MLR, NLR, PLR	Unstable(n=57)	Controls(n=57)	P value	Test statistical value
Monocyte to high-density lipoprotein (HDL) ratio (MHR)				
Mean ± SD	0.010 ± 0.011	0.004 ± 0.004	<.0001^§^	1194.5
Range	0.001-0.060	0.001-0.017		
Monocyte to lymphocyte ratio (MLR)				
Mean ± SD	0.16 ± 0.15	0.06 ± 0.06	<.0001^§^	1047
Range	0.01-0.89	0.01-0.31		
Neutrophil to lymphocyte ratio (NLR)				
Mean ± SD	2.01 ± 1.06	1.35 ± 0.36	<.0001^§^	1198
Range	0.01-6.8	0.66-2.65		
Platelet to lymphocyte ratio (PLR)				
Mean ± SD	119.9 ± 48.39	85.77 ± 30	<.0001^§^	1127
Range	42.5-315	36.98-168.86		

**Table 9:** Comparison of MHR, MLR, NLR, and PLR between stable vitiligo patients and age and gender-matched controls § Mann-Whitney test

MHR, MLR, NLR, PLR	Stable (n=15)	Controls (n=15)	P value	Test statistical value
Monocyte to high-density lipoprotein (HDL) ratio (MHR)				
Mean ± SD	0.008 ± 0.005	0.005 ± 0.003	0.225^§^	83.5
Range	0.001-0.016	0.002-0.012		
Monocyte to lymphocyte ratio (MLR)				
Mean ± SD	0.12 ± 0.08	0.09 ± 0.06	0.455^§^	94.5
Range	0.03-0.27	0.03-0.23		
Neutrophil to lymphocyte ratio (NLR)				
Mean ± SD	1.53 ± 0.56	1.45 ± 0.4	0.709^§^	103.5
Range	0.74-2.77	0.82-2.17		
Platelet to lymphocyte ratio (PLR)				
Mean ± SD	95.92 ± 18.79	95.66 ± 28.89	0.468^§^	95
Range	53-134.01	51.51-148.15		

Only PLR was significantly higher in unstable vitiligo patients when compared to stable vitiligo patients (Table 10).

**Table 10:** Comparison of MHR, MLR, NLR, and PLR between stable and unstable vitiligo patients § Mann-Whitney test

MHR, MLR, NLR, PLR	Stable (n=15)	Unstable (n=57)	P value	Test statistical value
Monocyte to high-density lipoprotein (HDL) ratio (MHR)				
Mean ± SD	0.008 ± 0.01	0.01 ± 0.01	0.846^§^	413.5
Range	0.001-0.016	0.001-0.06		
Monocyte to lymphocyte ratio (MLR)				
Mean ± SD	0.12 ± 0.08	0.16 ± 0.15	0.346^§^	359.5
Range	0.03-0.27	0.01-0.89		
Neutrophil to lymphocyte ratio (NLR)				
Mean ± SD	1.53 ± 0.56	2.01 ± 1.06	0.079^§^	301
Range	0.74-2.77	0.01-6.8		
Platelet to lymphocyte ratio (PLR)				
Mean ± SD	95.92 ± 18.79	119.9 ± 48.39	0.046^§^	283.5
Range	53-134.01	42.5-315		

**Figure 1:**
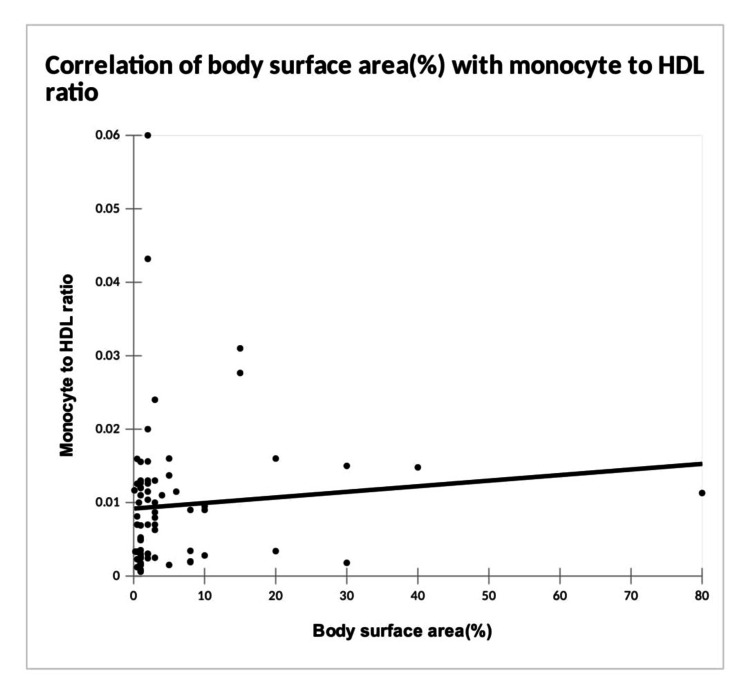
Correlation of body surface area (%) with monocyte to high-density lipoprotein (HDL) ratio (MHR) Correlation test: Spearman’s rank correlation, correlation coefficient = 0.24, P value = 0.043

There was a significant positive correlation between percentage body surface area involved and MHR (Figure 1).

However, no correlation was noticed between other ratios and the percentage body surface area involved (Table 11). There was no significant correlation between VETI and MHR, MLR, NLR, and PLR.

**Table 11:** Correlation of body surface area (%) with MHR, MLR, NLR, and PLR among vitiligo patients Spearman's rank correlation coefficient MHR: monocyte to high-density lipoprotein ratio; MLR: monocyte to lymphocyte ratio; NLR: neutrophil to lymphocyte ratio; PLR: platelet to lymphocyte ratio

Variables	Monocyte to HDL ratio	Monocyte to lymphocyte ratio	Neutrophil to lymphocyte ratio	Platelet to lymphocyte ratio
Body surface area (%)				
Correlation coefficient	0.240	0.221	0.101	-0.073
P value	0.043	0.063	0.399	0.542

Out of 72 vitiligo patients, 19 were started on systemic treatment as follows: 11 patients (58%) received systemic steroids in combination with either Azathioprine, Tofacitinib, oral Puvasol, or narrowband ultraviolet B (NBUVB) phototherapy. Four patients (21%) received systemic steroids alone, 3 patients (17%) were started on NBUVB phototherapy alone, and 1 patient (4%) received only cyclosporine. All the ratios were compared for these 19 patients before and after 1 month of treatment. There was no statistically significant reduction in these ratios, but there was a statistically significant reduction in mean VETI after one month of systemic treatment (p = 0.002).

## Discussion

Vitiligo is a chronic condition with multiple factors affecting its pathogenesis. Among these, oxidative stress and systemic inflammation play a crucial role in the progression of the disease. Various markers of oxidative stress in vitiligo have been studied earlier, but studies on markers like MHR, MLR, NLR, and PLR in vitiligo are few.

MHL, MLR, NLR, and PLR have been used as indicators of oxidative stress in various systemic and dermatological diseases.

Monocytes are the main sources of proinflammatory and oxidative cytokines. HDL inhibits the oxidation of low-density lipoprotein (LDL) and prevents its negative effect on the endothelium, thereby showing anti-inflammatory and antioxidant properties. The inflammatory process usually increases monocytes and decreases lymphocytes. So, MHR and MLR have been used as markers for systemic inflammation and oxidative stress in many autoimmune disorders, cardiovascular diseases, malignancies, and tuberculosis [1].

Neutrophils are potent sources of neutrophil elastase, matrix metalloproteinase (MMP), and cytokines. Lymphocytes have anti-inflammatory properties. Thereby, NLR is a reliable indicator of inflammation [1].

Platelets are also involved in inflammatory reactions and immune responses. PLR, mean platelet volume(MPV), and plateletcrit (PCT) can be used as markers for inflammatory diseases.

Kanbay et al., in a cohort study done on chronic kidney disease patients, suggested that high MHR is a bad prognostic factor for cardiovascular events in patients with chronic kidney disease [15]. Acikgoz et al. found that MHR can be a marker of inflammation and an early predictor of vascular involvement in Behcet’s disease [9]. He had conducted a case-control study with 60 cases and 50 controls and found that MHR was significantly raised in cases compared to controls. Sirin MC et al. used these markers to assess the inflammation in psoriasis, and it was seen that MHR and NLR were raised in psoriatic patients compared to controls. The psoriasis area and severity index (PASI) score was positively correlated with MHR, and higher MHR values were seen in patients with PASI >10 [8]. Patients with acne vulgaris had elevated levels of HDL and NLR in a case-control study conducted by Turkmend et al., but there was no significant difference observed in the other CBC parameters [10]. MHR was found to be considerably higher in lichen planus patients in a retrospective study by Temiz et al. [11]. Additionally, in cases of oral lichen planus, the oral disease severity score showed a correlation with MHR. Therefore, ratios based on complete blood count and HDL have been used as markers of inflammation and oxidative stress in a few dermatological disorders, but the results are inconsistent.

In our study, most vitiligo patients belonged to the age group of 21 to 30 years (27.78%), with a mean age of 38.64 years, comparable to the study by Temel et al. [16].

There was a slight male predominance in our study, which was in contrast to a previous study [1], with equal gender preponderance. The mean disease duration was found to be 5.55 years, which was comparable to a previous study [17]. The majority of our patients had unstable vitiligo, and among them, the most common VIDA score was 4+. This can be explained by the fact that vitiligo patients usually seek medical care when they develop new lesions or experience an increase in preexisting lesions.

In our study, most of the patients gave a history of alternative treatment followed by topical steroids, tacrolimus, topical and oral PUVASOL (topical psoralen plus Ultraviolet A obtained by solar light), and systemic immunosuppressants. This was similar to the findings of Sarma et al., which also showed a significant proportion of patients availing alternative treatment, probably pointing to the low patient satisfaction with available treatment options in vitiligo and lack of counselling for them at diagnosis [18].

The mean percentage of body surface area involved was 5.7%, which was comparable to the studies by Mahajan VK et al. and Sarma et al. [17,18]. Probably, even patients with few lesions feel compelled to seek medical attention because of the social stigma.

The most commonly involved sites were the limbs, which finding was similar to the findings of Mahajan VK et al. [17]. This was followed by the trunk, mucosa, and head in decreasing order of frequency. Mucosal involvement was seen in 40.28% cases, and most of them had only oral mucosa involvement. This predominance of oral mucosal involvement has also been observed in previous studies by Sarma et al. [18]. The most common pattern of vitiligo seen in our study was generalized, followed by acral, acrofacial, unclassified mucosal type, segmental, focal, and universal. The predominance of generalized vitiligo was consistent with the previous study by Mahajan et al. [17].

Our review of previously published data on the relationship between MHR, MLR, NLR, and PLR as markers of oxidative stress in vitiligo revealed that just two previous studies have examined such a correlation [1,16], and only a single Indian study has assessed the change in NLR and PLR after treatment of vitiligo so far [19]. The previous two studies on these markers in vitiligo patients by Demirbas et al. and Temel B et al. were conducted in Turkey, with a similar study design and comparable age and gender distribution [1,16].

Our study found a significant weak positive correlation between the VETI and VIDA scores. This was similar to the observation in a study by Awal G et al., where they found a positive correlation between VIDA and VASI scores [20], which is also a quantitative score measuring the extent of disease, similar to VETI.

We found an insignificant weak positive correlation of VETI with MHR, MLR, and NLR, while there was a weak negative correlation with PLR. However, in the study conducted by Demirbas et al., there was a significant positive correlation of these ratios with VETI except NLR (Table 12) [1].

**Table 12:** Comparison of the present study with previous studies S: significant; I: insignificant; MHR: monocyte to high-density lipoprotein ratio; MLR: monocyte to lymphocyte ratio; NLR: neutrophil to lymphocyte ratio; PLR: platelet to lymphocyte ratio

Study	Study design	MHR of cases compared to controls	MLR of cases compared to controls	NLR of cases compared to controls	PLR of cases compared to controls	Correlation with VETI
Demirbas et al. [1]	Case control, n=180	S	S	I	S	Positive correlation with MHR, MLR, PLR
Temel et al. [16]	Case control, n=69	S	I	I	I	Not studied
Solak et al. [21]	Case control: Localized = 50, Generalized = 43 Controls = 50	Not studied	Not studied	S	Not studied	Not studied
Present study	Case control, n = 72	S	S	S	S	I

This could be due to differences in ethnicity, age, and gender distribution between our study population and the population studied by Demirbas et al., which was in Turkey [1].

In our study, there was a significant positive correlation between the percentage of body surface area involved and MHR. This was consistent with the study conducted by Temel B et al., where both MHR and MLR were significantly higher in patients with generalized vitiligo compared to patients with localized vitiligo [16], even though in our study, only MHR was found to be significant. Therefore, the burden of oxidative stress could be higher in vitiligo patients with extensive involvement.

Solak et al., in a similar study, observed that the NLR was significantly higher in generalized vitiligo compared to the control and localized vitiligo groups (Table 12) [21]. Generalized vitiligo included acrofacial, vulgaris, and mixed types of vitiligo, and localized vitiligo included the focal and mucosal types of vitiligo.

In our study, we found that there were statistically significantly higher values of MHR, MLR, NLR, and PLR in vitiligo patients as compared to controls, similar to the observations seen in the study by Demirbas et al. [1], except NLR, which was not found to be significant. In the study by Temel B et al., only MHR was significantly higher in vitiligo patients compared to controls, while other ratios were higher in the patient group but not significant (Table 12) [16].

However, only PLR was significantly higher in unstable patients when compared to stable patients. These discrepant results could be due to the higher number of unstable cases (79.17%), compared to stable cases (20.83%), and the difference in the extent and activity of disease among patients. There was no significant difference in these markers between stable vitiligo patients and controls, suggesting that these markers could be used as markers of disease activity in vitiligo. There is a lack of studies comparing these markers between the stable and unstable groups of vitiligo patients; this was not done by Demirbas et al. or Solak et al. [1,21].

The analysis and comparison of our results with the two previous Turkish studies indicate that MHR could be a reliable and consistent marker of oxidative stress in vitiligo, while further studies are needed to validate the other markers.

In our study, we followed up 19 patients who were started on systemic treatment for one month, and these markers and VETI were reassessed and compared with baseline values. It was found that there was a significant reduction in VETI from the baseline after one month of systemic treatment. There are no previous studies using the VETI score to assess the treatment response to the best of our knowledge.

We also compared the pre- and post-treatment values of MHR, MLR, NLR, and PLR for those patients on systemic treatment after one month, which didn’t show any significant reduction. In a study by Taru Garg et al. on vitiligo patients, change in NLR and PLR was assessed after 3 months of daily 0.1% tacrolimus and thrice weekly NBUVB phototherapy [19]. They demonstrated a reduction from the baseline NLR and PLR, but it was insignificant, consistent with our study. Currently, other studies on the effect of systemic treatment on these ratios in vitiligo are lacking. Probably, a one-month duration was too short for a follow-up, as most of the systemic treatments take longer to reveal any significant effects. Further, the pre- and post-treatment comparison of oxidative stress markers becomes valid only when the treatment is uniform.

Limitations

Our sample size was relatively small. There was an unequal distribution of unstable and stable vitiligo patients, and the duration of follow-up after systemic treatment was shorter. Further, these ratios can be affected by other factors like smoking, recent surgery, trauma, and other inflammatory diseases like chronic cholecystitis, nephritis, bronchitis, and gout.

## Conclusions

MHR, MLR, NLR, and PLR are novel inflammatory markers that have been studied in various systemic and a few dermatological diseases. MHR and possibly MLR, NLR, and PLR are valuable markers in assessing oxidative stress in vitiligo, specifically in unstable vitiligo patients. PLR can be considered as a marker to determine the stability of vitiligo, which may help in guiding the treatment, as unstable vitiligo patients often require systemic treatment. Further studies with stratification of stable and unstable vitiligo patients for the assessment of these markers are indicated to assess the usefulness of these ratios in demonstrating an association with lack of stability. Similarly, a longer treatment duration would be required to reassess the reduction of these markers on treatment for vitiligo patients.
